# Dynamic flow priming programs allow tuning up the cell layers properties for engineered vascular graft

**DOI:** 10.1038/s41598-021-94023-9

**Published:** 2021-07-19

**Authors:** Kazutomo Baba, Andrey Mikhailov, Yoshiyuki Sankai

**Affiliations:** 1 Graduate School of Systems and Information Engineering, University of Tsukuba, 1-1-1 Tennodai, Tsukuba, Ibaraki 305-8573 Japan; 2 Center for Cybernics Research, University of Tsukuba, Tsukuba, Japan; 3 Faculty of Engineering, Information and Systems, University of Tsukuba, Tsukuba, Japan

**Keywords:** Biomaterials, Regenerative medicine, Tissue engineering

## Abstract

Tissue engineered vascular grafts (TEVG) are potentially clear from ethical and epidemiological concerns sources for reconstructive surgery for small diameter blood vessels replacement. Here, we proposed a novel method to create three-layered TEVG on biocompatible glass fiber scaffolds starting from flat sheet state into tubular shape and to train the resulting tissue by our developed bioreactor system. Constructed tubular tissues were matured and trained under 3 types of individual flow programs, and their mechanical and biological properties were analyzed. Training in the bioreactor significantly increased the tissue burst pressure resistance (up to 18 kPa) comparing to untrained tissue. Fluorescent imaging and histological examination of trained vascular tissue revealed that each cell layer has its own individual response to training flow rates. Histological analysis suggested reverse relationship between tissue thickness and shear stress, and the thickness variation profiles were individual between all three types of cell layers. Concluding: a three-layered tissue structure similar to physiological can be assembled by seeding different cell types in succession; the following training of the formed tissue with increasing flow in a bioreactor is effective for promoting cell survival, improving pressure resistance, and cell layer formation of desired properties.

## Introduction

Until recently, small artificial vascular prostheses, less than 6 mm in diameter, could not be used clinically due to lack of their antithrombotic and infection resistance and high chance of early occlusion^1–4^. When arteriosclerotic vascular disorders including ischemic heart disease, peripheral artery disease, or similar conditions occur, in some cases catheter treatment or stenting may not be possible, then surgical revascularization such as coronary artery bypass grafting or lower limb bypass surgery is required. Regular treatment of choice is utilizing the patient's own blood vessels, such as the internal thoracic artery or the great saphenous vein^5^. However, often in the patients with arterial disease, other blood vessels in their body are affected by stenosis as well, which makes it difficult to harvest a healthy vein necessary for bypass surgery^6^.

Vascular graft therapy based on tissue engineering is a treatment option in such cases^7^. Since tissue engineered vascular graft (TEVG) have good potential to replace and repair demanded areas, several approaches to construct TEVG were suggested so far^8^. Self-organization methods such as cell-sheet, micro tissue aggregation, or 3D bio-printing are scaffold-free methods utilizing only the cells^9–11^. They have common advantage of biocompatibility, yet each method has its own limitations. Cell sheets method allows mimicking the layered structure of blood vessels by stacking one sheet at a time. However, the stacking of dense sheets of cells disrupts the supply of nutrients and oxygen to the interior layer^12^. Micro tissue aggregation is reliable method for tissue formation in required size and we previously developed a method and the bioreactor for its implementation to form tubular tissue of arbitrary diameter and length^13^. However, it is challenging to control the self-assembly of the cells, and no one has yet been able to reproduce a physiological vessel with a correct three-layered structure by this approach. 3D bio-printing could make layer structure in principle, however, since gravity applied in the height direction, the stacked length is actually limited whether a bio-printer handles individual cells or spheroids as a single dispensing unit^14^. Another challenge of 3D cell printing is cell viability due to extrusion pressure^15^.

The clinical application of TEVG requires reconstruction of vascular function based on the three-layered tissue structure, as well as scalability in size and the strength to withstand blood flow pulses and surgery suturing, but it is still difficult and timely to satisfy all these requirements with just self-assembly approaches in current technologies. In this study, rather than relying on self-assembly only, we proposed a method to form a vascular layer structure close to physiological with biocompatible glass fibers. We also developed a novel bioreactor and housing devices which allowed to form the cell layers, to mature the layer structure, and to train the assembled vascular tissues with various perfusion flows. In addition, we examined mechanical and biological features of the constructed tubular tissue and the relationship between the properties of the composed layers and the training flow rate inside the bioreactor.

## Results

### Layer structure formation during sheet culture period

**Figure 1:**
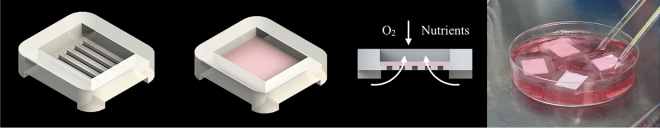
Silicone frame design for a glass fiber sheet set up and cell layer formation. Far right: Actual frames with glass fiber scaffold preconditioned in culture media. A part of the image was created by the rendering function of SOLIDWORKS 2018.

**Figure 2:**
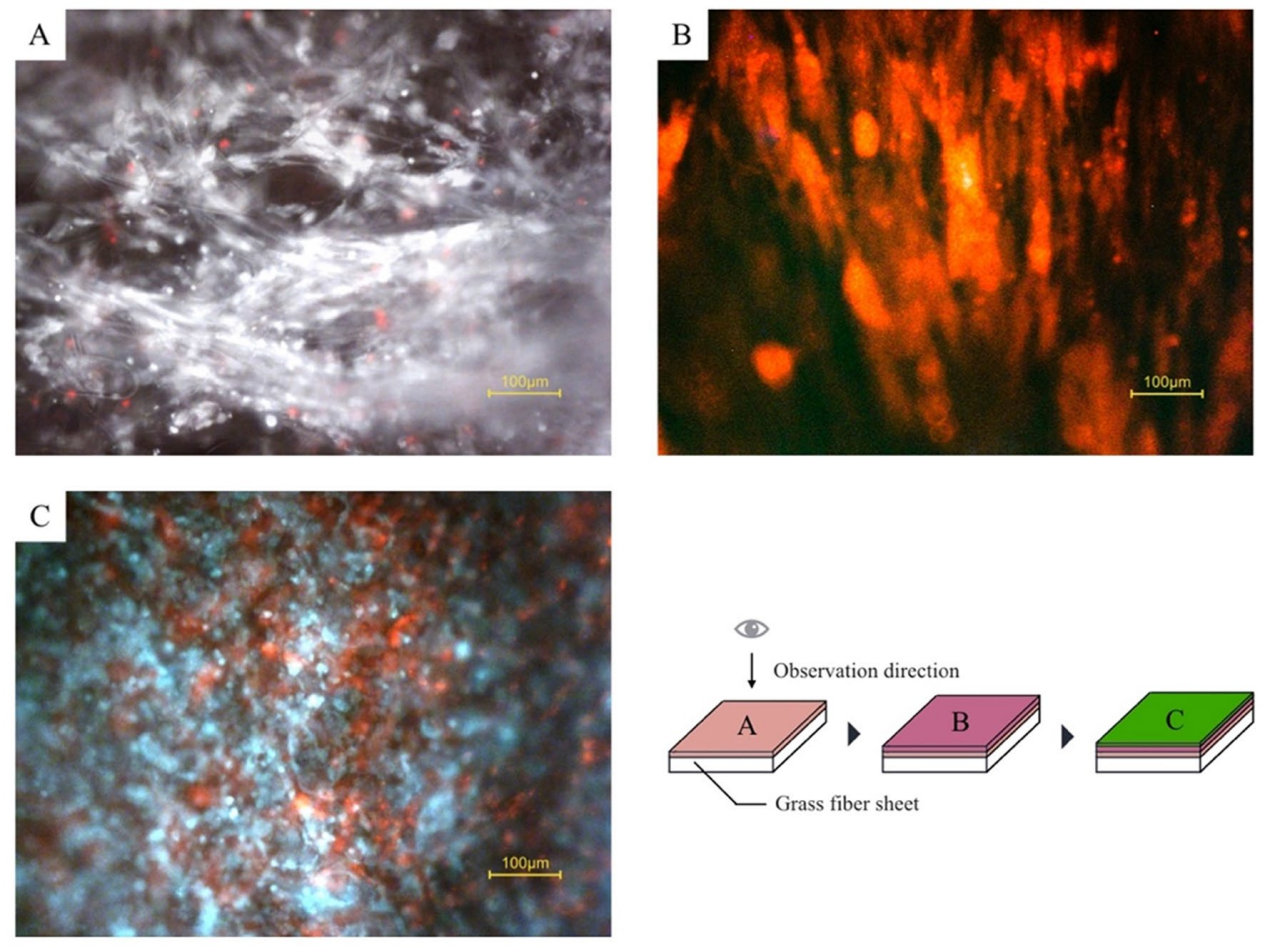
Formation of the tissue layers on a glass fiber sheet at each maturation step during sheet culture period. (**A**) First layer, white: fibroblasts stained by Calcein-AM, red: dead cells stained by ethidium, (**B**) Second layers, red fluorescent: RFP-SMCs. Fibroblasts on the background are non-fluorescent, (**C**) Third layers, red: RFP-SMCs, teal: GFP-HUVECs with automatic white balance. Each picture was taken under inverted fluorescent microscope after completing tissue maturation steps at weekly intervals. (**C**) Endothelial cell layer (blue) is in front of the smooth muscle cell layer (red), and the cells are close to be evenly distributed on the glass fiber.

Cells were seeded onto sterilized glass fiber sheets placed into silicon frames of our design (Fig. 1) allowing media access from both sides of the sheets. Layers of cells on glass fiber sheets were assembled in following succession: fibroblasts (NHDFc), red fluorescent protein (RFP) expressing Human Aortic Smooth Muscle Cells (hASMCs), and green fluorescent protein (GFP) expressing Human Umbilical Vein Endothelial Cells (HUVECs). Fibroblast showed invasive growth with penetration into the material (Fig. 2A); hASMCs and HUVEC formed cell layers on top of the previously seeded cells (Fig. 2B,C).

### Cell density and viability

**Figure 3:**
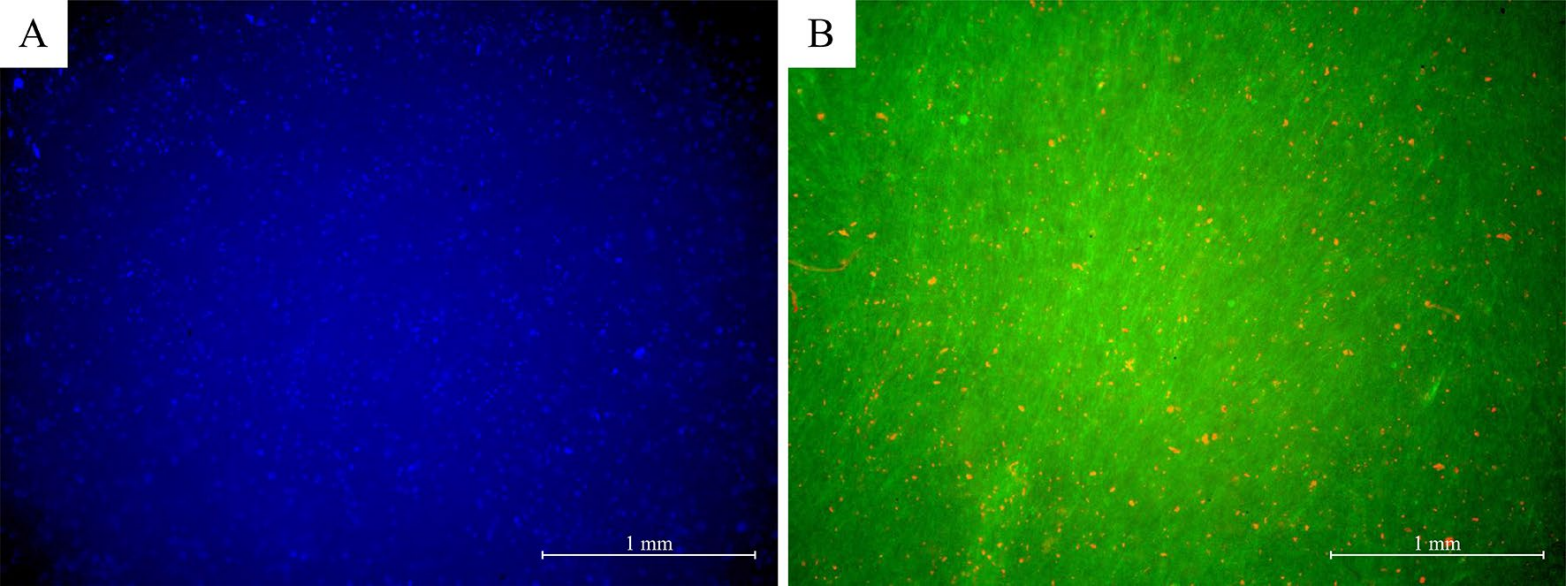
Survival of cells in the formed tissues on a GF sheet after maturation in the developed bioreactor. (**A**) DAPI stained nuclei, (**B**) Staining with Calcein-AM and Ethidium.

Count of DAPI stained nuclei (Fig. 3A) of cells in NHDFc layer was compared quantitatively with results of staining with Calcein AM and Ethidium (Fig. 3B). Five random field of view images were taken from two independent samples. Cell layer borders in 3D tissue image were overlapping, so staining with Calcein AM could be used for the visualization of live cells prevalence only, but not for the calculation of cell number. Table 1 shows the number of cells averaged from 5 images, their density, and cell viability calculated from these results. Most of the cells’ nuclei were negative for Ethidium staining and the survival rate was estimated at 95.8 ± 1.4%.

**Table 1:** Number of cells, density and survival rate defined by Calcein AM/Ethidium staining.

Total number of cells in the field of view	Number of cells/1 mm^2^	Survival rate (%)
1226 ± 31	123 ± 3	95.8 ± 1.4

### The changes of the mechanical strength of glass fiber during tissue formation

**Figure 4:**
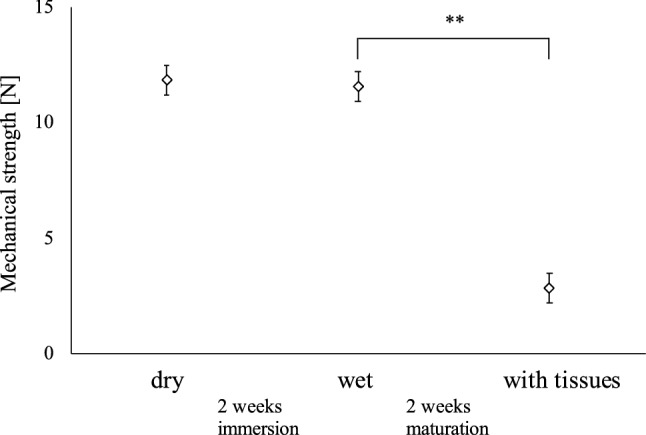
Tensile strength measurement of the glass fiber sheet before and after tissue formation with the developed tensile tester device. **p < 0.01 by two-tailed t-test with an alpha level of 0.05. The error bar relates to the standard deviation of the mean.

**Figure 5:**
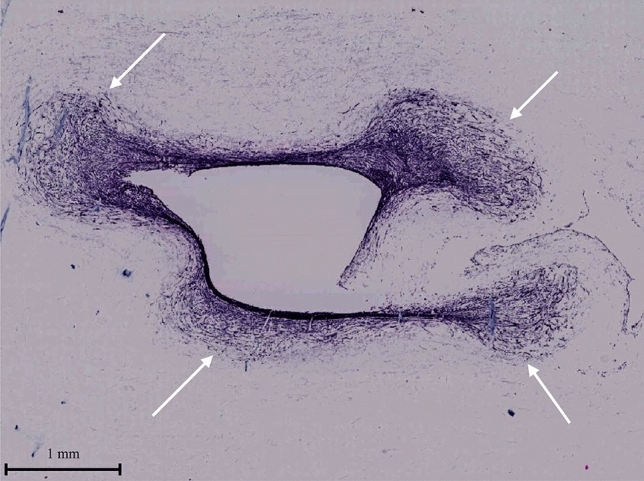
Infiltration of fibroblasts into a glass fiber. White arrows: invasive growth areas.

There was little change in the tensile strength of the glass fiber itself before and after autoclaving with subsequent 2 weeks long immersion in the culture media (p = 0.83) as shown in Fig. 4. On the other hand, glass fiber sheets lost the tensile strength significantly (p < 0.01) after seeding and growing the fibroblasts. The tensile strength of the glass fiber sheets decreased from 11.56 ± 1.98 N in the dry state to 2.83 ± 0.76 N in fibroblast-infiltrated samples. The depth of the infiltration area after fibroblast culture (shown by the white arrows in Fig. 5) was examined, the cells infiltrated up to 1.26–1.48 mm from the inner surface of the scaffold, which is 70–80% of the thickness of the swollen scaffold (approximately 1.8 mm). We suggest that the invasive growth of the fibroblasts into the depth of the glass fiber may disturb connections between the fibers and impaired its strength.

### Burst pressure resistance

**Figure 6:**
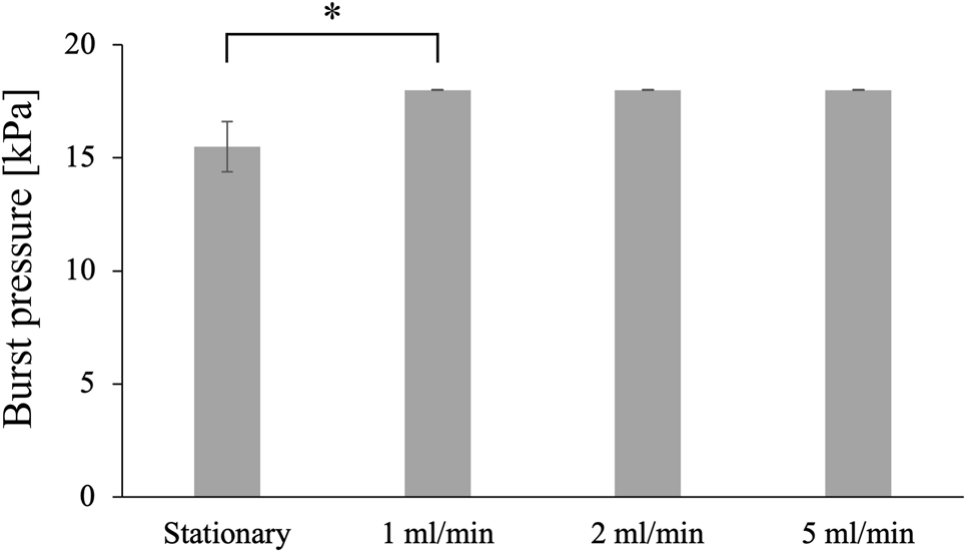
Burst pressure resistance of the tissue trained at different flow rate. *p < 0.05 by two-tailed t-test with an alpha level of 0.05. The error bar represents the standard deviation of the mean.

The results of the burst pressure measurements are shown in Fig. 6. All tissues trained with the bioreactor were found to withstand the maximum test pressure specified by ANSI/AAMI VP20:1994^16^ and ISO7198:2016^17^. The burst pressure resistance for all the trained tissues showed statistically significant difference compared to the stationary culture (p = 0.03).

### Tubular structure formation and tissue removal

**Figure 7:**
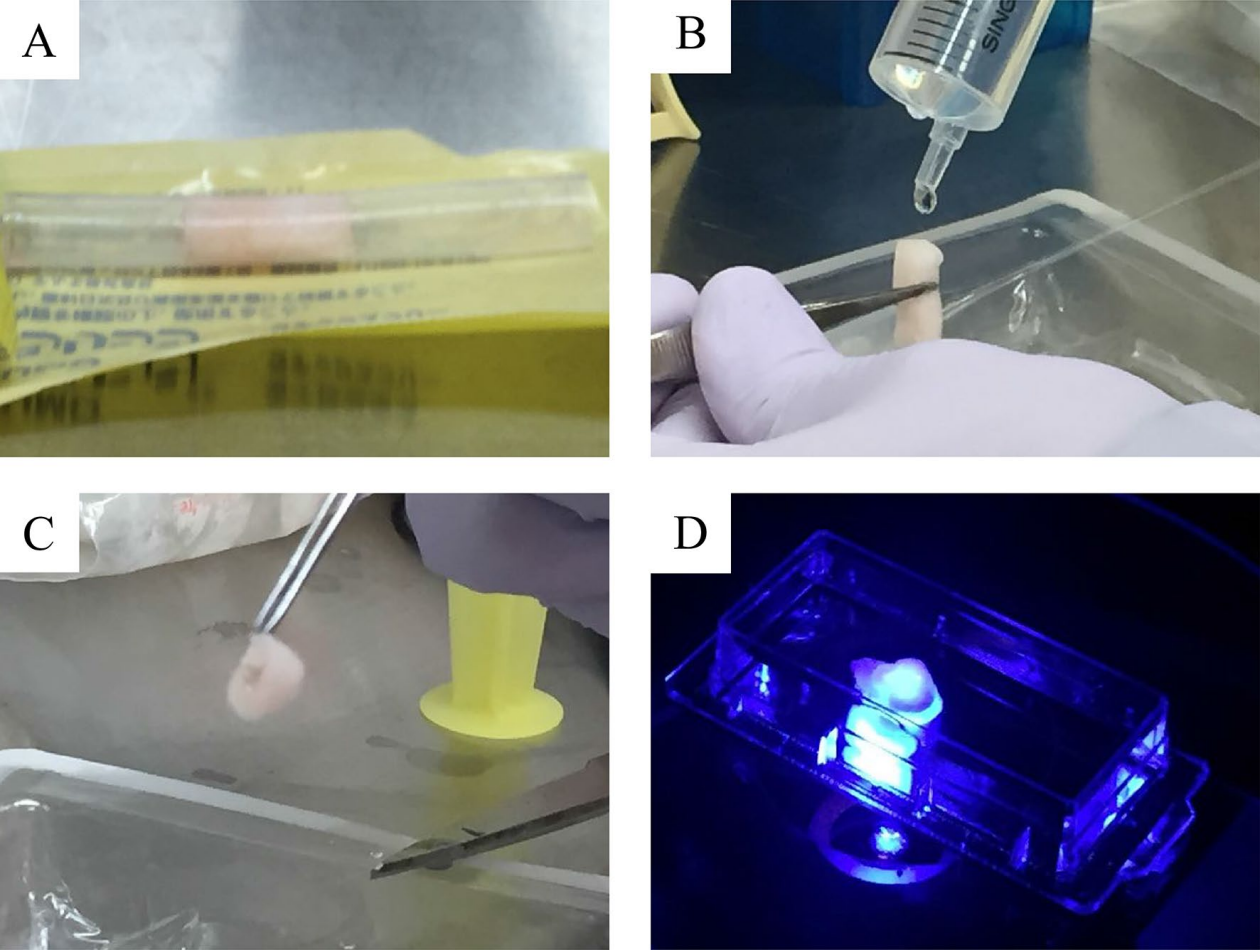
Matured blood vessels after training in the bioreactor. The tubular shape could be retained without much damage by some experimental procedures. (**A**) Extraction, (**B**) Washing with PBS, (**C**) Cutting/Slicing, (**D**) Observation.

**Figure 8:**
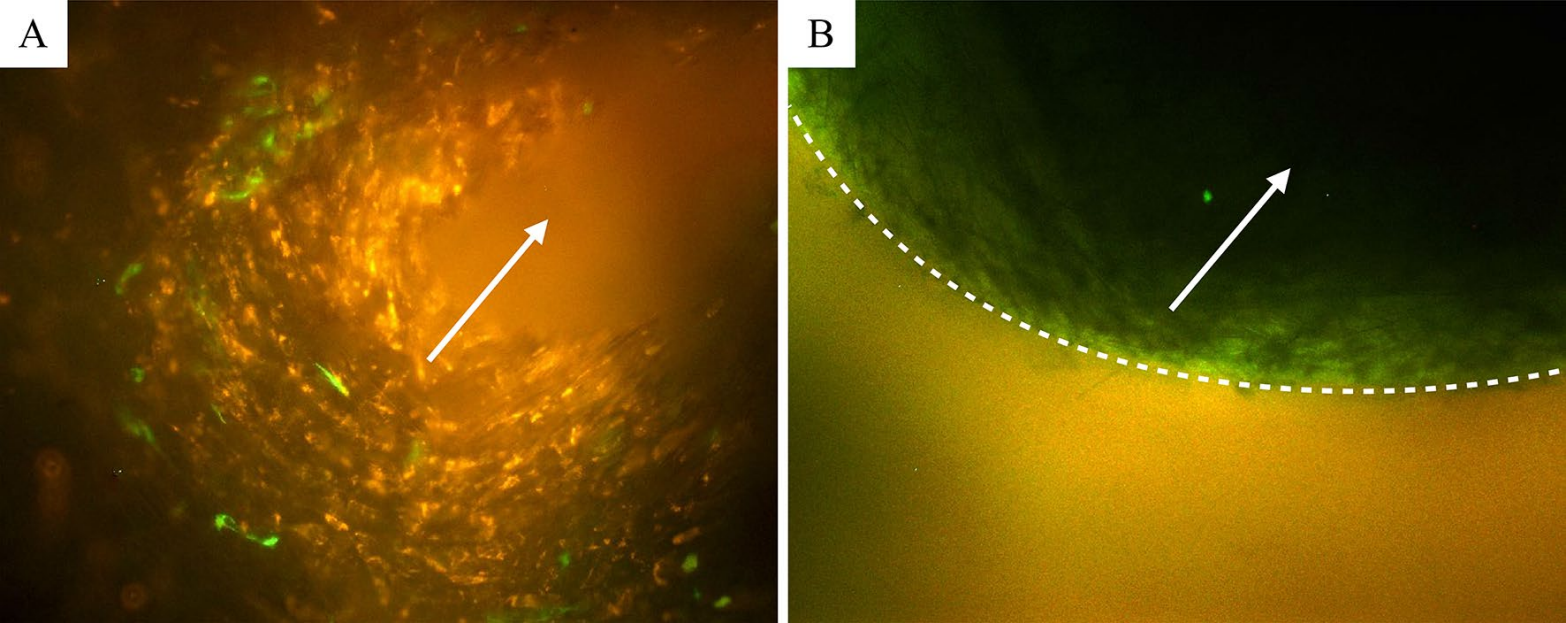
Cross sections of three-layer blood vessels. (**A**) 5 ml/min composite of 20 images with different focal planes from the front to the back of the freshly formed tissue. (**B**) 1 ml/min higher magnification image with wall exposure, white dotted line: defined separation of two layers. Both; red: RFP-SMC, green: GFP-HUVEC, white arrow: flow direction.

Glass fiber sheets with 3 cell layers were successfully rounded into a tube shape by the proposed method with the help of sterilized metal rod 3.5 mm in diameter and the structure was stabilized with surgical suture. Tubular proto-tissues were placed into 4-channel bioreactor of our design and matured by individual training flow programs. Complete cycle of training and maturation took 2 weeks. Although, some experimental procedures such as removal of the formed tissue from the system, cutting it with scissors, washing the tissue with PBS, and pinching with tweezers were challenging to perform, we visually confirmed in most of the cases that the tubular shape could be retained without much damage (Fig. 7). After crosscut, the tissues were placed into transparent plastic dishes, and live fluorescence imaging of the vascular tissue was performed in the cross-sectional direction. We could observe that the shape of the tissue was close to tubular, and the green fluorescent endothelial cells could be seen inside the red fluorescent smooth muscle cells layer (Fig. 8A). Stronger layer of endothelial cells could be seen in samples with different training program as on Fig. 8B, where endothelial cells spread more evenly on the hASMCs layer and the two layers had clear border.

### Histological analysis and shear stress calculations

**Figure 9:**
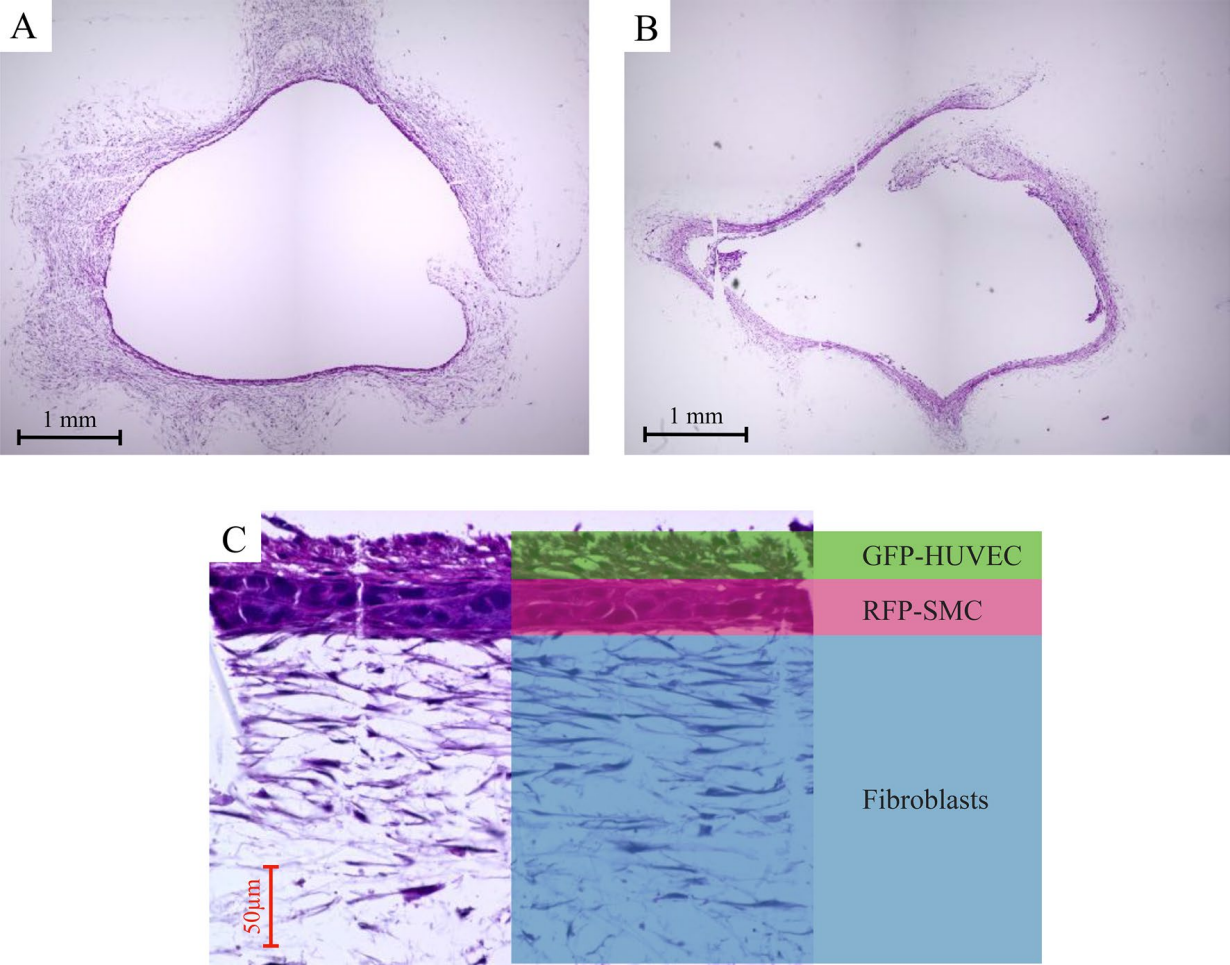
(**A**) Overview of a representative cross section of a 1 ml/min three-layered blood vessel stained by HE. (**B**) A 5 ml/min three-layered blood vessel. (**C**) Enlarged view of three layers. Example of thickness calculation of each cell layer.

**Figure 10:**
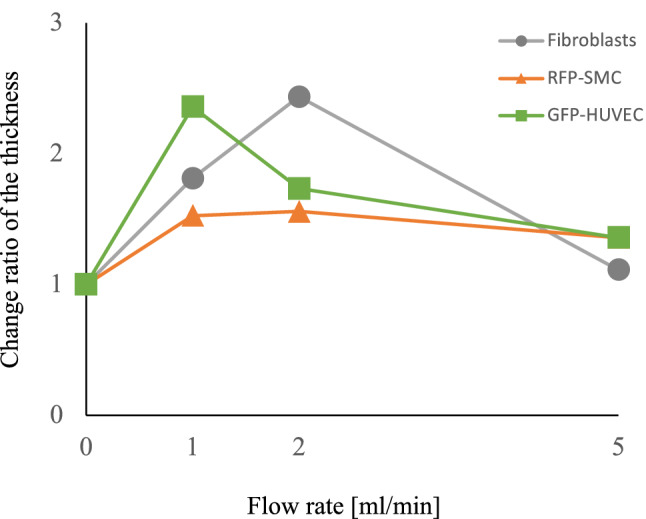
Trends of layer thickness change for the three types of cells at each flow rate compared with static culture.

21 cross-cut synthetic vascular tissue samples were stained with HE to confirm histologically the fusion and organization of each cell layer and to calculate each layer’s thickness (Fig. 9). The effect of the perfusion flow rate on the maturation of tissue layer structure was analyzed. Individual trends of the cell layer’s thickness change at each flow rate compared to the base thickness of the stationary culture are presented in Fig. 10. The thickness of all cell layers increased when the flow was applied compared to the stationary culture. The same situation occurred to all types of cells comprising our tissues, suggesting that sustained nutrient and oxygen delivery by the bioreactor and associated mechanical training allowed the cells to increase in number and area. On the other hand, the further increase in tissue thickness of all cell types was abolished at higher flow rates.

**Figure 11:**
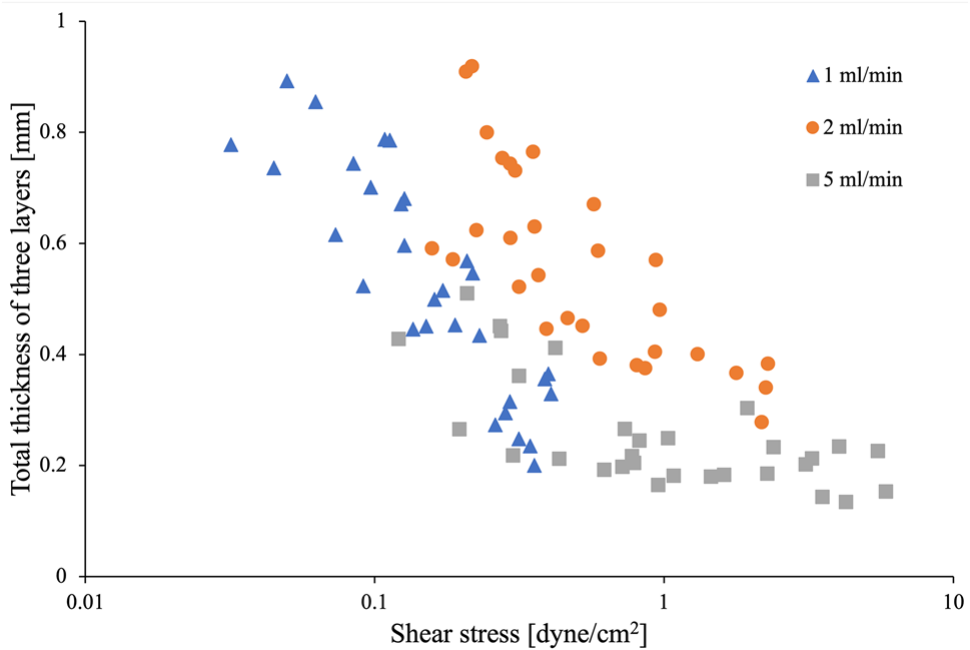
Relationship between the total thickness of the three layers in matured tissue (i.e., infiltration depth into the scaffold) and the shear stress.

The shear stress was calculated at each flow rate and its relationship to the tissue thickness was analyzed. The results are shown in Fig. 11. The 30 samples used for calculation at each flow rate had the following range of shear stress values: 0.03–0.41 dyne/cm^2^ at 1 ml/min, 0.16–2.29 dyne/cm^2^ at 2 ml/min, and 0.12–5.85 dyne/cm^2^ at 5 ml/min. Tissue thickness showed a similar trend at 1 ml/min and 2 ml/min flow rates, with a decreasing trend as shear stress increased. At 5 ml/min, tissue thickness showed a trend similar to other flow rates, although, the tissue thickness remained minimum and at relatively constant level as shear stress increased.

TUNEL staining was used to investigate the cell survival rate upon the bioreactor culture. Compared to stationary culture, there was statistically insignificant increase in cell viability at all flow rates where training was applied (Table 2).

**Table 2:** Survival rate after bioreactor training at each flow rate defined by TUNEL staining. p > 0.05 by One-way ANOVA with an alpha level of 0.05.

Flow rate (ml/min)	Survival rate (%)
0 (stationary culture)	82.1 ± 7.1
1	95.5 ± 1.5
2	85.6 ± 0.7
5	90.3 ± 5.5

## Discussion

Small diameter conventional artificial vascular grafts have major limitations with respect to thrombosis, infection, and biocompatibility. Development of small-diameter TEVGs using regenerative medicine and tissue engineering approaches is an area of extensive reserch^8,18–20^. The supply of nutrients and oxygen has always been recognized as an important issue in the development of three-dimensional tissue^21^. Moreover, since normal blood vessels are constantly exposed to blood flow, it is particularly important to consider the effect of mechanical stimulation during in vitro culture^22^. Bioreactor-based culture systems hold the potential to provide a testing platform that is more predictable of a whole tissue response by providing more physiologically relevant conditions comparing to customarily used two- and three-dimensional cultures^23,24^. Here we proposed a novel method to create three-layered TEVG with biocompatible glass fibers as supporting scaffold and to train a tissue by the developed bioreactor system. Constructed tubular tissues were matured and trained under 3 types of individual flow, and we confirmed their mechanical and biological features.

In current study, we utilized glass fiber sheet as the scaffold. The glass fiber has good biocompatibility and it could be applied safely in animals^25,26^. In the previous work, the CNTs-interfaced phosphate glass fiber scaffolds provided support with good viability for PC12 cells. Authors suggested possible role of ionic extracts from the glass fibers in stimulating cell metabolism^27^. Other group has reported that bioactive glass fiber scaffolds were suitable substrates for the growth and differentiation of osteoblasts^28^. Our selected glass fiber sheets satisfied all of the following specifications required for constructing three layered blood vessel; (1) biocompatibility—to avoid foreign body reactions and rejection, (2) negligible cytotoxicity—to ensure proper cell survival, (3) surface allowing cell adhesion—to facilitate mechanical support, (4) porosity for nutrients and oxygen supply, (5) flexibility permitting sheet-to-tube folding, and (6) sufficient material strength to withstand the experimental manipulation. The glass fiber sheets we used consist of a porous material with a particle retention capacity of 2.7 µm, which allow seeding the cell layers with suspension of the individual cells. Material has sufficient permeability for the culture medium, and it has the flexibility to be formed into a tube shape (Fig. 7).

Similar attempt combining cell sheet engineering and electrospinning technology was performed, and bioreactor preconditioned SMC sheet-combined vascular scaffold maintained high cell viability (95.9 ± 2.7%), phenotypes and improved cellular infiltration, as well as desired mechanical properties^9^. We confirmed that the cells were uniformly attached and spread along the fibrous direction (Fig. 2). The survival rate of the cells was at level of 95.8%. From these results, we suggested that porous glass fiber sheet is useful as a biocompatible material for tissue preparation. Certainly, it lacks biodegradability, so future developments should be aimed for use of biodegradable glasses^26,29,30^ which were not available for us in a form of fiber filters at the time of our study.

Besides glasses, other materials were used for cellular support in TEVG. TEVG with biodegradable polyglycolic acid (PGA) fibers implanted subcutaneously into nude mice showed typical blood vessel structure and completely degraded scaffold in 6–8 weeks after^31^. However, subcutaneously formed tissues are not exposed to hydrodynamic environment, and since endothelial cells have been added to the implant afterwards it is difficult to isolate the effects of scaffold lysis from mechanism of cell infiltration and formation of the layered structures. Our system allows complete multilayered tissue maturation prior to implantation, tracking the changes in cell behavior following changes in physiological conditions. Bilayered small diameter vascular scaffold fabrication approach was developed by combining thermally induced phase separation and electrospinning technology, and the prepared scaffold scored in the tensile tests^32^. Although, the structure of the cell layer after implantation was not similar in physiology to the blood vessel. Developed silicon frame allowed us to seed the cells over glass fiber scaffold without losing majority of the cells and to stack the layers in succession. Uniform cell distribution and sequential layered structure meet the physiological structure requirements. Another work employed crosslinked electrospun gelatin scaffolds of specific fiber layer orientation, and high suture retention strength was achieved in the range of 1.8–1.94 N for wet acellular scaffolds, same or better than that for fresh saphenous vein^33^. The tensile strength measurements of our scaffold showed that there was little change in strength between dry and soaked material, however tensile strength was dramatically reduced after cell culture possibly due to the fibroblast invasion to maximum 70–80% depth into the scaffold. Nevertheless, our vascular tissue with glass fiber scaffold has a tensile strength of 2.83 N, which is sufficient to withstand the use of sutures. In fact, no heavy damage was observed during the operation of rounding and tying tissues with sutures within the proposed method. The burst pressure test results suggest that training with the bioreactor improves pressure resistance at least to the range of blood pressure stipulated by ISO7198:2016^17^.

In our study, formation of the three-layered structure on the glass fiber seeded with differentiated primary human cells of three types was confirmed, and the assembled layer order was maintained during all incubation period in the tube shape. As an alternative to primary cells, differentiated iPS cells can provide an attractive cell source for constructing TEVGs using the sheet engineering technique^34^. Recent article reported generation of hiPSC-derived TEVGs with mechanical strength comparable to native vessels used in arterial bypass grafts by utilizing biodegradable scaffolds, incremental pulsatile stretching, and optimized culture conditions^35^. On the other hand, the technology to assemble the sheets of endothelial cells and fibroblasts derived from iPS cells with SMCs has not yet been realized, it seems to be still difficult to form a multilayered vascular structure using a pipe-like scaffold (including molds). The sheet-to-tube folding using glass fiber scaffold with multiple cell types gave the advantage of creating a three-layered structure in intended order and maintaining it after formation of the tube.

Regarding cell organization in the scaffold, each layer thickness tended to decrease as the flow rate reached maximum specific for the cell types and the shear stress increased. Some publications investigated the detachment of endothelial cells due to shear stress^36,37^, although our range of applied shear stress was relatively low, and direct comparison is challenging. Several works have reported that endothelial cells and smooth muscle cells decrease their proliferation depending on shear stress in the laminar flow^38–40^, and the response of the cell layers to shear stress at 5 ml/min flow rate in our culture is consistent with these reports. The tendency was observed for smooth muscle cells, but not as drastic as for endothelial cells. In our case, the response of smooth muscle cells to shear stress seems lower than endothelial cells.

The histological analysis revealed that the cells infiltrating the glass fiber were mostly fibroblasts, which means that some fibroblasts were located far from the inner surface of the vessel wall where the shear stress acted. This suggests that some factors other than shear stress could contribute to their thickness change. Previously, systematic computer simulations study of different types of scaffolds in the dynamic bioreactor showed predicted oxygen concentration profiles at the center of the pore in a fibrous scaffold, and predicted cell front propagation distance from the seeded surface of the scaffold^41^. Simulations showed that cells fill the pores of the porous scaffold over a period of days, and the oxygen concentration decreases on a log-like scale as the distance from the seeded surface increases^41^.

We initially utilized dermal fibroblasts in our study. Vascular fibroblasts differed from the dermal morphologically at two-dimensional culture, they also showed slower grow rate and limited replicative potential. On top of that, dermal fibroblasts are more available for syngeneic tissue engineering, since sacrificing the small area of skin is less invasive, that sacrificing the same biomass of the vascular tissue from the patient. Nevertheless, after cultivation in bioreactor the tri-layered tissue with vascular fibroblasts showed similar histological structure and mechanical properties. Since the dermal fibroblasts in our study had superior properties at cell culture level, we believe the use of dermal fibroblasts for synthetic tissue construction is justified at this point. Our comparison of the flow rate and histological structure of the tissue points to invasion of fibroblast cell into glass fiber scaffold in area of slower flow and, perhaps, lower mass rate exchange between circulated media and forming tissue. Is it an influence of lower oxygen tension, nutrients concentration or waste products insufficient removal? Interplay between cell oxygen tension and physiological responses (migration in particular) is not known in details and some data are controversial. For example, cultured L929 fibroblasts under hypoxic conditions (1% O_2_) demonstrated enhanced cell spreading, decrease of single cell migration, and a decline of cell motility^42^. 24 h hypoxia exposure govern fibrotic responses in cardiac fibroblasts: their proliferation, secretion of inflammatory and pro-fibrotic cytokines in culture supernatants; myofibroblast differentiation^43^. Indirectly attributed to migration is finding that low oxygen tension coupled with macromolecular crowding significantly accelerate extracellular matrix deposition and the development of scaffold-free tissue-like modules. Interestingly, fibroblasts exhibited the highest metabolic activity at slightly hypoxic conditions—2% oxygen tension and more extracellular matrix proteins—collagens type I, V, and VI and fibronectin were deposited at 2% oxygen tension, as opposed to 0.5% and 20%^44^.

On the other hand, some of the observed effects might be attributed to effective exchange between media and trained tissue in area with higher flow rate. It was demonstrated that high glucose conditions (25 mM d-glucose, or about 4500 mg/L in fresh media we used) inhibited cell migration when compared to low glucose concentration^45^. Comparing behavior of several types of the cells at different oxygen and glucose concentrations authors have found that survival of fibroblasts was higher at slightly hypoxic conditions (5%) comparing to both severe hypoxia (0.1%) or normoxia (21% of oxygen in gas mix)^46^.

From these results, some insights and limitations for future development can be suggested. A three-layered tissue structure can be created by seeding the cells in sequence using a sheet-like porous glass fiber scaffold giving the advantage that the physiological structure TEVG can be formed as intended. In addition, it has mechanical properties that allow it to be easily rounded and maintain its shape. Our newly developed bioreactor was capable of simultaneous comparative experiments with individual flow rate settings, and training of tissue formation with increased flow by the bioreactor was effective in promoting cell survival, and improved burst pressure resistance of the tissue. Tested, nearly equal ratio between the cell types corresponds closer to the structure of veins; to assemble an artery several folds increase in smooth muscle cell layer thickness expected to be necessary to achieve the burst resistance level capable of withstanding the elevated blood pressure scenario. At that time, selecting individually and providing the proper flow rate is important factor for the cell layer formation close to physiological. The interrelationship between the cell layer (especially fibroblast), shear stress, and mass exchange when culturing three-layered TEVGs are revealed at initial level so far and requires further study.

## Methods

### Cells

We used 3 types of cells normally comprising blood vessels: Fibroblasts (NHDFc, C-12302, and HAoAF, C-12380 PromoCell, Heidelberg, Germany), Smooth muscle cells (hASMCs, cAP-0026RFP, Angio-Proteomie, Boston, USA), and Endothelial cells (HUVECs, cAP-0001GFP, Angio-Proteomie, Boston, USA). Frozen stock of each types of the cells was thawed and scaled up in appropriate media: D-MEM (High Glucose with Phenol Red and Sodium Pyruvate, StemSure, Wako) supplemented with 10% of fetal calf serum for smooth muscle cells and fibroblasts and Endothelial media (Endothelial Cell Growth Medium, PromoCell) for endothelial cells in 75 cm^2^ flasks (Primaria, Corning, NC, USA). Before co-culture cells were adapted for 1:1 mix of the above media for 72 h. For layer formation cells were detached from the surface with Trypsin/EDTA solution (Wako, Japan), and washed with its cultured media filtered through 1.2 µm membrane filter.

### Scaffold

We used glass fiber filters (WHATMAN GRADE GF/D, GE Healthcare) for cell support and formation of the tubular shapes after. The filter had an average predicted pore size of 2.7 µm, a fiber diameter of 3 µm, a basis weight of 121 g/m^2^, a typical thickness of 675 µm which can swell up to 2–3 times by water absorption, and factory specified wet burst of 0.3 psi (= 2068 Pa). Filters were cut into pieces (16.5 × 20 mm), sterilized by autoclaving and soaked with cell culture media 24-h prior the cell seeding.

### The silicon frame design for cell seeding on glass fiber scaffold

The cells in suspension have similar density to their culture media, so forming the cell layer on flat sheet in bigger Petri dish led to escape of sizeable part of seeded cells from intended surface. Therefore, a PDMS silicon frame was developed to accommodate the glass fiber sheet (Fig. 1). The size of the glass fiber sheet was set 16.5 × 20 mm which can form a tubular shape with 4 mm inner diameter, 6 mm outer diameter, and 20 mm length when it is bent into the tube. When the mold was submerged inside the dish, the culture medium could permeate from both top and bottom.

### Biocompatibility

Since we used glass fiber supporting scaffold, which was not specifically designed for cell attachment, ensuring its biocompatibility was necessary prior to further experiments. Evaluation of the biocompatibility was done by seeding NHDFc on sterilized filters and measuring cell viability after 7 days growth with LIVE/DEAD Viability/Cytotoxicity Kit (MOLECULAR PROBES/Thermo-Fischer). Since projections of cell bodies on 3D scaffold are heavily overlapped, we used DAPI staining to count the total cell number and staining with Ethidium homodimer to assess the number of dead cells with damaged membrane (necrotic and late apoptotic cells). For each measurement we used 5 separate field of view, calculation was done with ImageJ software^47^.

### Tensile strength measurements

**Figure 12:**
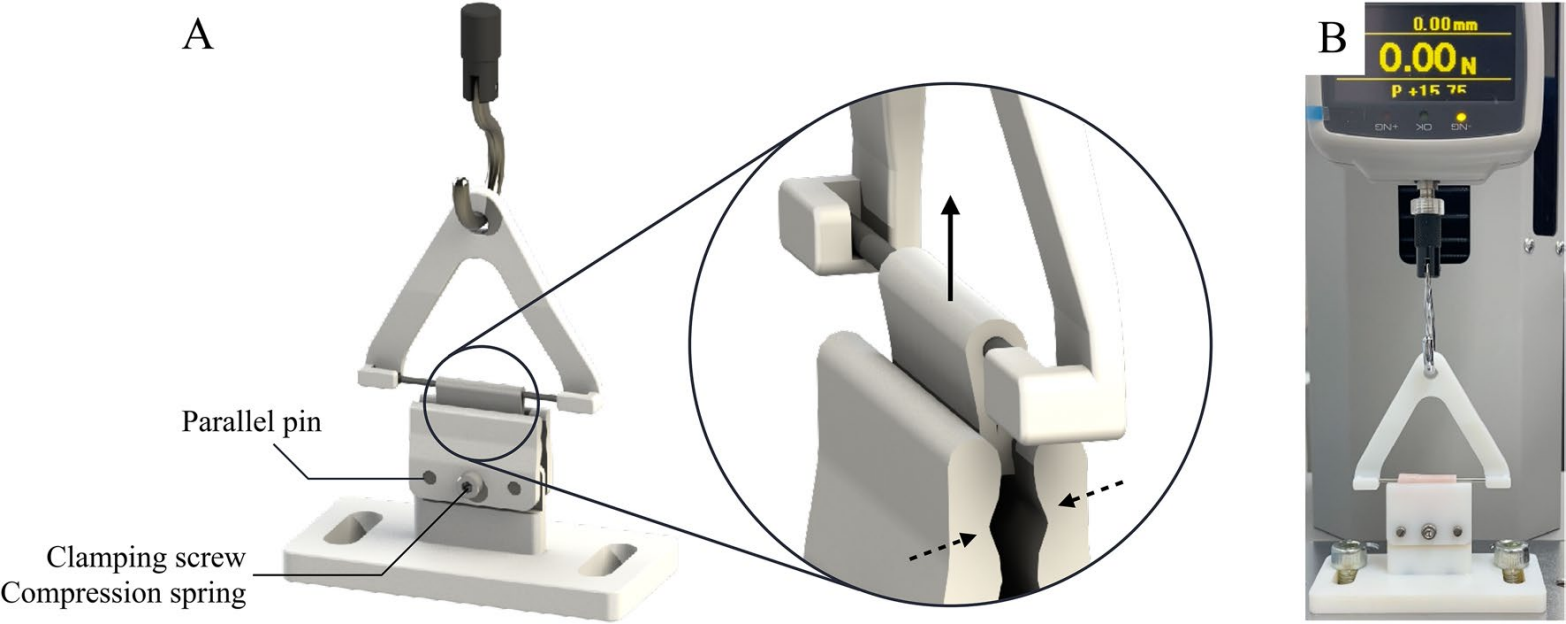
Tensile strength measurement tester device. (**A**) The developed tensile holder. (**B**) Overall appearance of the device assembled with the Load/Displacement Measurement Unit and digital force gauge. A part of the image was created by the rendering function of SOLIDWORKS 2018.

We developed a holder for comparative measurement of dry and wet glass fiber sheets before and after tissue formation (Fig. 12A). Tensile strength of glass fiber was measured using the holder of our own design, Load/Displacement Measurement Unit (FSA-1KE-20N, IMADA, Japan), and digital force gauge (ZTA-20N, IMADA, Japan) shown in Fig. 12B, in order to verify whether the strength of glass fiber was changed by infiltration of the culture medium. The filter (n = 4) was wrapped around the axel in terminal holders and pulling force applied with a steady rate of 50 mm/min until the break point according to the Sect. 8.3.1 in ANSI/AAMI VP20:1994^16^. We measured 3 sets of filters: factory dry, sterilized and kept wet, and filters with cells.

### Three-layered structure formation of tissue engineered blood vessels

**Figure 13:**
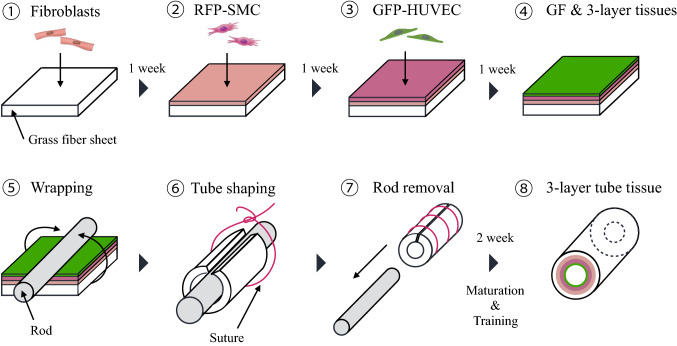
Construction method of three-layer blood vessel using glass fiber as a scaffold. (1) Fibroblast seeding. (2) Smooth muscle cell seeding on top of fibroblast layer. (3) Endothelial cells seeding onto two-layer smooth muscle cells/fibroblast structure. (4) Layer structure conditioning and tissue maturation. (5) Wrapping the flat sheet around the metal rod to form tubular shape. (6) Holding the tube shape with surgical suture. (7) Rod removal/central channel formation. (8) Formed three-layer blood vessel before tissue maturation and training.

To mimic physiological structure of blood vessel we propose a method in which the culture process is divided into stages as illustrated in Fig. 13. First, the fibroblast (NHDFc or HAoAF) layer, which is the outer layer of vessels, was seeded on top of the pretreated glass fiber. We used about 10^7^ cells for each layer. One week after the media was changed and the next layer was formed with hASMCs, and after another week the same operation repeated with HUVECs. For maturation of three-layered tissue, we used additional 7 days with two media changes during that period.

### Bioreactor and training system

**Figure 14:**
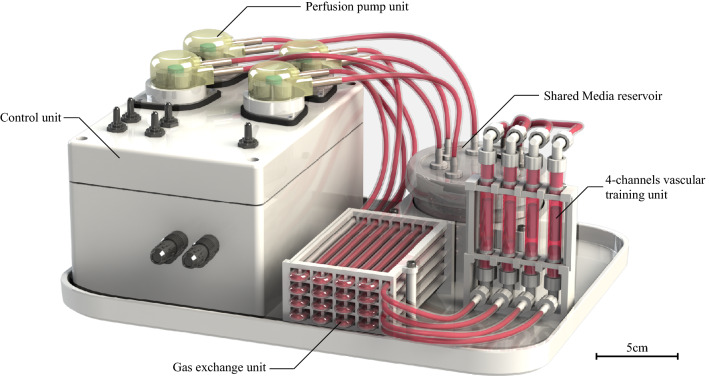
The overview of the 4-channel bioreactor for maturation of proto-tissues into the trained vascular tissue by individual training flow programs. Scale bar = 5 cm. A part of the image was created by the rendering function of SOLIDWORKS 2018.

Our bioreactor system consists of a control unit, an autoclavable perfusion pump unit (WPM2-P3EA-CP, Welco), a gas exchange unit, 4-channels vascular training unit, and shared media reservoir (Fig. 14). Every component except the control unit was sterilized by autoclaving. The size of entire system is 30 cm length, 20 cm width, and 17 cm height, and whole device was installed inside a CO_2_ incubator during entire culture period.

The control unit consists of a main circuit board which regulate the flow rate and reads sensor values, stepper motors, motor drivers (L6470, STMicroelectronics), temperature and humidity sensor, polycarbonate housing, and a rubber sealing frame. This unit adjust a flow rate of the perfusion pump unit between 0 and 48 ml/min. Four stepper motors have daisy-chained each other and allow to support individual flow control or batch synchronous control. Since each circulation pathway of the media is mechanically independent, it allows to perform and analyze the cultivations under individual flow conditions. The flow rate and its procedure of changing up to the target value can be set by the user via an external serial communication port.

The gas exchange unit consists of four silicon tubes (3 mm inner diameter) of about 2 m long wrapped around the supporting stand and they were connected between the shared reservoir and each training unit. The media passing through this unit delivers oxygen to cells due to reasonable gas permeability of silicon as well as stabilize carbon dioxide level necessary for maintenance of pH buffer capacity of the culture media.

4-channels vascular training unit consist of silicon tube housings (8 mm diameter), several luer connectors, and the supporting stand. The culture medium transported from the pump passes mainly through the inner side of the tubular tissue installed in the silicone housing. Media flows allow to mature proto-tissues and to give the shear stress for the training of cell-to-cell adhesion and extra cellular matrix adaptation. A slight gap between the outside of the tubular tissue and the housing provided flow of the culture medium, supplying oxygen and nutrients to the cells through the glass fiber scaffold material.

### Tubular shape formation by developed auxiliary device

**Figure 15:**
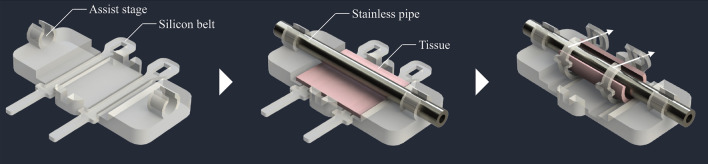
An auxiliary device for aseptic and reproducible tissue wrapping on glass fiber. A part of the image was created by the rendering function of SOLIDWORKS 2018.

After confirming by life fluorescent microscopy of the flipped over filter that the smooth muscle cells and the endothelial cells layers have been formed, we returned filters in upright position and placed them in fresh media. Forming of continuously round shape for glass fiber filter with attached cell layers is challenging. Therefore, we developed an auxiliary device for ensuring safety and reproducibility of the glass fiber wrapping (Fig. 15). In this device, a sheet is placed on the stage first, then a metal pipe (3.5 mm diameter) is placed on the sheet and fixed to both tube ends, and the tubular shape could be initialized and maintained by wrapping the glass fiber sheet with two silicon belts. At this state, the glass fiber sheet around the tube is tied and fixed at three of more points with surgical sutures. With this assistive device, the operation could be completed in 5–10 min per one tissue to avoid the tissue drying.

The tissues then were placed into sterilized silicone tube housing and connected into the bioreactor system we have developed. Culture medium circulated through the housing for two weeks with changing of 50% of the media to the fresh one every 120 h. As a result, tissues were subjected to shear stress and tension from the fluid in the similar way as blood vessels in the body, which was expected to promote cell-to-cell adhesion and a reinforcing mechanical stress response.

### Maturation and training of the tissue in the developed bioreactor

**Figure 16:**
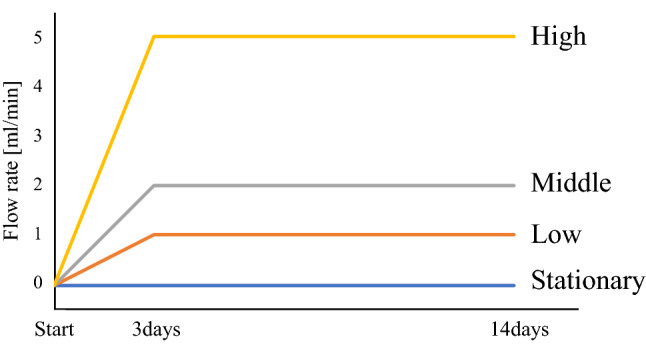
Training flow programs.

Four equal glass fiber sheets with 3 cell layers were prepared for each experiment. They were placed inside the silicon tube housings in the system, sealed with connectors, and the individual flow supplied from the individually programmed pumps to reach 1.0, 2.0, and 5.0 ml/min during 72 h, and then the maturation and training was continued at the steady flow rate for 2 more weeks (Fig. 16).

### Live imaging

The distribution of cells in the inner part of the trained tubular tissues was assessed by help of fluorescent proteins expressed in smooth muscle cells (red) and endothelial cells (green). Tubular tissues with surrounding glass fiber scaffold were crosscut, washed with warm PBS and placed in Petri dishes under the layer of PBS on inverted fluorescence microscope (Nikon eclipses Ti). Several images were acquired with different depth of focus and restored by Adobe Photoshop into single combined image.

### Pressurized burst strength measurements

Burst pressure tests against water pressure were conducted according to ANSI/ISO^16,17^. During the test, the vascular tissue was sandwiched between O-rings (cell layers facing up) from above and below, and the hydrostatic pressure was gradually increased to a maximum of 18 kPa (= 135 mmHg) while measuring pressure with a sensor (GC31-364, NAGANO KEIKI CO., LTD., Japan) delivering corresponding DC current to the analog-to-digital converter (ATmega328P, Microchip Technology Inc., USA), and the burst pressure was registered by camera when the water droplets started to appear. Measurements were performed at six different locations of the unfolded samples for each cultivation flow rates. The areas with severely damaged or inconsistent cell layers were excluded from the final calculations.

### Histological evaluation

The tissues removed from bioreactor housing were washed with warm PBS, and fixed in 4% paraformaldehyde in phosphate buffer for 20 min at 37 °C. The fixed tissues were sent to external processing (New Histo Science Laboratory Co. Ltd., Tokyo, Japan) for sectioning and staining. HE and TUNEL stained slides were photographed (BZ-9000 BioRevo microscope, Keyence Corp., Japan) and thickness of different cell layers estimated in quadruplicates for each image.

HE images of tissue samples trained at flow rates of 0, 1, and 5 ml/min (n = 3 for each flow rate) were used to examine the shear stress at 10 selected points in the vessel. Shear stress is expressed as

$$\tau = 4\mu Q/\pi r^{3} ,$$

where μ is the viscosity, Q is the flow rate and r is the vessel radius (the distance from the center of lumen and a tissue border). Then, individual cell layers and their total tissue thickness at the same point was also calculated.

### Statistical analysis

The method of Student’s t test was used to determine the significance of difference in each parameter between any 2 selected groups. The method of One-way ANOVA for multiple comparisons was used to determine the significance of difference in multiple groups. A difference was considered statistically significant at p < 0.05.

## References

[1] Isenberg BC, Williams C, Tranquillo RT (2006). Small-diameter artificial arteries engineered in vitro. Circ. Res..

[2] Teebken OE, Haverich A (2002). Tissue engineering of small diameter vascular grafts. Eur. J. Vasc. Endovasc. Surg..

[3] Zilla P, Bezuidenhout D, Human P (2007). Prosthetic vascular grafts: Wrong models, wrong questions and no healing. Biomaterials.

[4] Baquey C (2008). Developments towards tissue-arterial substitutes. Tissue Eng..

[5] Faries PL (2000). A comparative study of alternative conduits for lower extremity revascularization: All-autogenous conduit versus prosthetic grafts. J. Vasc. Surg..

[6] Gerhard-Herman MD (2017). 2016 AHA/ACC guideline on the management of patients with lower extremity peripheral artery disease: Executive Summary: A report of the American college of cardiology/American Heart Association task force on clinical practice guidelines. Circulation.

[7] Pashneh-Tala S, MacNeil S, Claeyssens F (2016). The tissue-engineered vascular graft—Past, present, and future. Tissue Eng. Part B Rev..

[8] Carrabba M, Madeddu P (2018). Current strategies for the manufacture of small size tissue engineering vascular grafts. Front. Bioeng. Biotechnol..

[9] Ahn H (2015). Engineered small diameter vascular grafts by combining cell sheet engineering and electrospinning technology. Acta Biomater..

[10] Kelm JM (2010). A novel concept for scaffold-free vessel tissue engineering: Self-assembly of microtissue building blocks. J. Biotechnol..

[11] Itoh M (2015). Scaffold-free tubular tissues created by a bio-3D printer undergo remodeling and endothelialization when implanted in rat aortae. PLoS One.

[12] Rademakers T, Horvath JM, van Blitterswijk CA, LaPointe VLS (2019). Oxygen and nutrient delivery in tissue engineering: Approaches to graft vascularization. J. Tissue Eng. Regen. Med..

[13] Baba K, Mikhailov A, Sankai Y (2018). Combined automated culture system for tubular structure assembly and maturation for vascular tissue engineering. J. Biomech. Sci. Eng..

[14] Duan B (2017). State-of-the-art review of 3D bioprinting for cardiovascular tissue engineering. Ann. Biomed. Eng..

[15] Thattaruparambil Raveendran N, Vaquette C, Meinert C, Samuel Ipe D, Ivanovski S (2019). Optimization of 3D bioprinting of periodontal ligament cells. Dent. Mater..

[16] Cardiovascular implants—Vascular graft prostheses. *Association for the Advancement of Medical Instrumentation ANSI/AAMI VP20:1994* (2000).

[17] Cardiovascular implants and extracorporeal systems—Vascular prostheses—Tubular vascular grafts and vascular patches. *International Organization for Standardization ISO7198:2016(E)* (2016).

[18] Ratcliffe A (2000). Tissue engineering of vascular grafts. Matrix Biol..

[19] Radke D (2018). Tissue engineering at the blood-contacting surface: A review of challenges and strategies in vascular graft development. Adv. Healthc. Mater..

[20] Niklason EL, Langer SR (1997). Advances in tissue engineering of blood vessels and other tissues. Transpl. Immunol..

[21] McMurtrey RJ (2016). Analytic models of oxygen and nutrient diffusion, metabolism dynamics, and architecture optimization in three-dimensional tissue constructs with applications and insights in cerebral organoids. Tissue Eng. Part C Methods.

[22] Song HHG, Rumma RT, Ozaki CK, Edelman ER, Chen CS (2018). Vascular tissue engineering: Progress, challenges, and clinical promise. Cell Stem Cell.

[23] Massai D (2020). Bioreactor platform for biomimetic culture and in situ monitoring of the mechanical response of in vitro engineered models of cardiac tissue. Front. Bioeng. Biotechnol..

[24] Peroglio M, Gaspar D, Zeugolis DI, Alini M (2018). Relevance of bioreactors and whole tissue cultures for the translation of new therapies to humans. J. Orthop. Res..

[25] Jeans LA, Gilchrist T, Healy D (2007). Peripheral nerve repair by means of a flexible biodegradable glass fibre wrap: A comparison with microsurgical epineurial repair. J. Plast. Reconstr. Aesthet. Surg..

[26] Starritt NE, Kettle SAJ, Glasby MA (2011). Sutureless repair of the facial nerve using biodegradable glass fabric. Laryngoscope.

[27] Ahn HS (2015). Carbon-nanotube-interfaced glass fiber scaffold for regeneration of transected sciatic nerve. Acta Biomater..

[28] Brown RF (2008). Growth and differentiation of osteoblastic cells on 13–93 bioactive glass fibers and scaffolds. Acta Biomater..

[29] Burova I (2019). A parameterised mathematical model to elucidate osteoblast cell growth in a phosphate-glass microcarrier culture. J. Tissue Eng..

[30] Colquhoun R, Tanner KE (2015). Mechanical behaviour of degradable phosphate glass fibres and composites—A review. Biomed. Mater..

[31] Shen G (2003). Tissue engineering of blood vessels with endothelial cells differentiated from mouse embryonic stem cells. Cell Res..

[32] Guo X (2019). Facile preparation of a controlled-release tubular scaffold for blood vessel implantation. J. Colloid Interface Sci..

[33] Elsayed Y, Lekakou C, Labeed F, Tomlins P (2016). Fabrication and characterisation of biomimetic, electrospun gelatin fibre scaffolds for tunica media-equivalent, tissue engineered vascular grafts. Mater. Sci. Eng. C.

[34] Hibino N (2012). Evaluation of the use of an induced puripotent stem cell sheet for the construction of tissue-engineered vascular grafts. J. Thorac. Cardiovasc. Surg..

[35] Luo J (2020). Tissue-engineered vascular grafts with advanced mechanical strength from human iPSCs. Cell Stem Cell.

[36] van Kooten TG (1994). Fluid shear induced endothelial cell detachment from glass—influence of adhesion time and shear stress. Med. Eng. Phys..

[37] Inoguchi H, Tanaka T, Maehara Y, Matsuda T (2007). The effect of gradually graded shear stress on the morphological integrity of a huvec-seeded compliant small-diameter vascular graft. Biomaterials.

[38] Cunningham KS, Gotlieb AI (2005). The role of shear stress in the pathogenesis of atherosclerosis. Lab. Investig..

[39] Liu SQ (1999). Focal expression of angiotensin II type 1 receptor and smooth muscle cell proliferation in the neointima of experimental vein grafts relation to eddy blood flow. Arterioscler. Thromb. Vasc. Biol..

[40] Akimoto S, Mitsumata M, Sasaguri T, Yoshida Y (2000). Laminar shear stress inhibits vascular endothelial cell proliferation by inducing cyclin-dependent kinase inhibitor p21 Sdi1/Cip1/Waf1. Circ. Res..

[41] Elsayed Y, Lekakou C, Tomlins P (2019). Modeling, simulations, and optimization of smooth muscle cell tissue engineering for the production of vascular grafts. Biotechnol. Bioeng..

[42] Vogler M (2013). Hypoxia modulates fibroblastic architecture, adhesion and migration: A role for HIF-1α in cofilin regulation and cytoplasmic actin distribution. PLoS ONE.

[43] Ugolini GS (2017). Human cardiac fibroblasts adaptive responses to controlled combined mechanical strain and oxygen changes in vitro. Elife.

[44] Kumar P, Satyam A, Cigognini D, Pandit A, Zeugolis DI (2018). Low oxygen tension and macromolecular crowding accelerate extracellular matrix deposition in human corneal fibroblast culture. J. Tissue Eng. Regen. Med..

[45] Lamers ML, Almeida MES, Vicente-Manzanares M, Horwitz AF, Santos MF (2011). High glucose-mediated oxidative stress impairs cell migration. PLoS One.

[46] Lafosse A, Dufeys C, Beauloye C, Horman S, Dufrane D (2016). Impact of hyperglycemia and low oxygen tension on adipose-derived stem cells compared with dermal fibroblasts and keratinocytes: Importance for wound healing in type 2 diabetes. PLoS One.

[47] Schneider CA, Rasband WS, Eliceiri KW (2012). NIH Image to ImageJ: 25 years of image analysis. Nat. Methods.

